# Quantifying overlap between the Deepwater Horizon oil spill and predicted bluefin tuna spawning habitat in the Gulf of Mexico

**DOI:** 10.1038/srep33824

**Published:** 2016-09-22

**Authors:** Elliott L. Hazen, Aaron B. Carlisle, Steven G. Wilson, James E. Ganong, Michael R. Castleton, Robert J. Schallert, Michael J. W. Stokesbury, Steven J. Bograd, Barbara A. Block

**Affiliations:** 1 Environmental Research Division, NOAA SWFSC, Monterey, California 93940, USA; 2 Department of Ecology and Evolutionary Biology, University of California Santa Cruz, Santa Cruz, California 95064, USA; 3 Hopkins Marine Station, Stanford University, Pacific Grove, California 93950, USA; 4 Department of Biology, Acadia University, Wolfville, Nova Scotia, B4P 2R6 Canada

## Abstract

Atlantic bluefin tuna (*Thunnus thynnus*) are distributed throughout the North Atlantic and are both economically valuable and heavily exploited. The fishery is currently managed as two spawning populations, with the GOM population being severely depleted for over 20 years. In April-August of 2010, the Deepwater Horizon oil spill released approximately 4 million barrels of oil into the GOM, with severe ecosystem and economic impacts. Acute oil exposure results in mortality of bluefin eggs and larvae, while chronic effects on spawning adults are less well understood. Here we used 16 years of electronic tagging data for 66 bluefin tuna to identify spawning events, to quantify habitat preferences, and to predict habitat use and oil exposure within Gulf of Mexico spawning grounds. More than 54,000 km^2^ (5%) of predicted spawning habitat within the US EEZ was oiled during the week of peak oil dispersal, with potentially lethal effects on eggs and larvae. Although the oil spill overlapped with a relatively small portion of predicted spawning habitat, the cumulative impact from oil, ocean warming and bycatch mortality on GOM spawning grounds may result in significant effects for a population that shows little evidence of rebuilding.

The Atlantic bluefin tuna (*Thunnus thynnus*) is a large endothermic, highly migratory species that is broadly distributed throughout the Atlantic Ocean from the waters off Greenland and Norway in the north to Argentina in the south^1^,^2^,^3^. Atlantic bluefin tuna are one of the most highly valued fish in the world which has led to the heavy exploitation of all three bluefin tuna species (Atlantic bluefin, southern bluefin *T. maccoyii*, Pacific bluefin *T. orientalis*)^4^,^5^. Atlantic bluefin tuna (ABFT) migrate seasonally between productive temperate and subpolar foraging habitats to warm subtropical habitats where they feed and spawn^3^,^6^,^7^,^8^. Historically, fish from the western population were hypothesized to spawn in the Gulf of Mexico (GOM) and those from the eastern population to spawn largely in the Mediterranean^3^,^9^,^10^,^11^,^12^. A recent study has shown spawning in Atlantic slope waters but it remains unclear how much this spawning area contributes to the western Atlantic bluefin tuna stock^13^. Both Mediterranean and GOM spawning populations are now known to mix and feed in waters off the western north Atlantic, particularly along the eastern seaboard of North America, while recent tagging, otolith michrochemistry, and genetic studies have shown that large adult fish feeding in the Gulf of St. Lawrence are largely fish from the western population^6^,^7^,^9^,^14^. Following feeding in the waters off the east coast of North America, fish from the western Atlantic stock migrate south to the GOM from November to August with a peak in spawning during the spring and early summer^6^,^12^,^15^,^16^.

Both the GOM and Mediterranean Atlantic bluefin tuna populations have been greatly reduced due to overexploitation, however the GOM population is smaller and has been more heavily impacted by overfishing than the Mediterranean population^17^. The GOM spawning stock biomass is 20% of 1970 levels and the ensuing 20-year rebuilding plan has not stemmed declines, highlighting the long term effects of fishing pressure on this depleted population^10^,^18^. Although no bluefin tuna are targeted in the US portion of the GOM due to existing fishery restrictions, they are caught as bycatch on yellowfin longline fisheries, prompting seasonal closures in the past two years^7^,^12^,^16^.

Bluefin tuna in the GOM spawn primarily in March – June in waters warmer than 24 °C yet often avoiding the warmest loop current waters, likely minimizing physiological stress and optimizing survival of spawned larvae^12^,^16^,^19^. Predictions of future warming scenarios for the GOM indicate that physiology may limit available spawning habitat unless tuna spawning shifts to earlier in the year^20^. Given the importance of the GOM as a spawning location for the western population^6^,^12^,^16^,^19^, cumulative effects from anthropogenic activities that impact this critical habitat during the spawning season need to be assessed.

On April 20^th^ 2010, the Deepwater Horizon (DWH) oil platform exploded in the northern GOM, releasing approximately 4 million barrels of crude oil into the ocean until the well was sealed in September 2010, resulting in one of the largest oil spills in history^21^,^22^. The shelf and slope waters of the northern GOM are a biodiverse area that serve as a spawning location for many pelagic fish species, including bluefin tuna, dolphinfish, marlin and swordfish^12^,^16^,^23^, in addition to supporting a variety of economically important commercial and recreational fisheries. Impacts of oil on the biology of higher trophic levels are particularly difficult to assess, as observed mortality is typically a significant underestimate, less than 10% of actual mortality^24^. Exposure of fish eggs and larvae to crude oil and weathered oil byproducts has a variety of deleterious, sublethal effects, including reduced growth rates, genotoxicity, impaired cardiac function, morphological malformations and premature hatching^25^,^26^,^27^,^28^,^29^. Less is known about impacts on juvenile or adult tunas. Given the depleted status of the GOM population of bluefin tuna^18^, their use of the GOM as a spawning area^16^, and the vulnerability of eggs and larvae to Polycyclic aromatic hydrocarbon contaminants^26^,^27^, the oil spill may have had significant effects on the health of the GOM population of bluefin tuna. Therefore, continued monitoring of adult tuna in addition to the 2010 year class is needed to understand population level effects.

Understanding the risks incurred by marine species in relation to anthropogenic stressors requires information on the habitat use and movements of marine animals as well as the spatial and temporal patterns of their stressors^30^,^31^. Published data on spawning events and larval distribution show a peak in May suggesting that multiple bluefin tuna life stages (eggs, larvae and adults) were likely exposed to oiled waters in the GOM^12^,^16^. In fact, two electronically tagged adult bluefin tuna exhibited putative spawning behaviors in the vicinity of the oil spill, spending several weeks in the waters around the Macondo well^7^. Using nekton surveys, Muhling *et al.*^11^ estimated that between 5–12% of larval bluefin tuna in the US EEZ were exposed to contaminated waters each week during the oil spill, although the bulk of spawning habitat and larvae were outside the area affected. Predicted adult bluefin habitat use in the GOM can be used to determine their potential exposure to contaminated waters, while the location and timing of spawning will determine the extent that eggs and larvae may be exposed to oil products. Hence, a more complete understanding of the potential impact that the oil spill had on bluefin tuna adults and larvae requires knowledge of how these fish use the GOM and where they spawn.

Here we use an extensive satellite and archival tag dataset from mature Atlantic bluefin tuna^7^ on GOM spawning grounds to model adult presence and spawning habitat within a generalized additive mixed modeling (GAMM) framework. In addition, we parameterized a Bayesian model to identify potential spawning events. Remotely-sensed oceanographic data were used to generate a spatially and temporally explicit estimate of where bluefin tuna were and where spawning occurred in the GOM during the DWH oil spill. We then combined habitat and spawning predictions with the spatial extent of the oil spill to quantify the overlap between bluefin tuna spawning habitat and oiled waters. Our approach provides both a time-varying and cumulative estimate of the impact of the DWH oil spill on adults and potentially multiple life history stages of bluefin tuna.

## Methods

A total of 125 bluefin tuna were satellite tagged in the southern Gulf of Saint Lawrence (GSL in the waters off Cape Breton Island, Nova Scotia) in September through October from 2007 to 2014. Tags were programmed to release during their late summer return to the GSL foraging ground in order to recover the tag after fully sampling the Gulf of Mexico. Post-release tags transmitted location via Argos satellites, and when possible tag recovery was attempted from a shore-based recovery team^7^. In addition, we also examined historical data from surgically implanted or externally attached archival tags deployed in the Gulf of Mexico and off Cape Hatteras and Morehead City, North Carolina from 1999–2005 (n = 24)^12^. All bluefin tuna were caught on rod and reel and tagged using two generations (MK10 and miniPATs) of Wildlife Computers pop-up satellite archival tags (PAT) over the course of the study. Light levels recorded by the tags were processed to generate daily estimates of latitude and longitude which were then matched sea surface temperature (SST) data from tag and remotely sensed data following Teo *et al.*^32^. Position estimates were refined using a probabilistic state space model (SSM) that incorporated maximum dive depth and bottom bathymetry and enabled quantification of the uncertainty associated with each daily position^6^,^33^,^34^. The model was validated with endpoint data from tagged Atlantic bluefin tuna (n = 72). For detailed information on tag preparation, programming, attachment, construction, and geolocation analyses see Block *et al.* and Wilson *et al.*^6^,^7^. All experimental protocols were carried out in accordance with relevant guidelines and regulations, and were approved by the Administrative Panel on Laboratory Animal Care of Stanford University and the Acadia University Animal Care Committee.

From the electronic tagging dataset, we examined 66 Atlantic bluefin tuna with tags recovered and that entered the GOM (which occurred between November and June) to examine residency and habitat preference (Supplemental Table 1). Bluefin were considered to have entered the GOM once they passed the 80.5° W meridian and remained within the GOM for six days. The resulting GOM bluefin tuna dataset encompassed 5272 tracking days with individual track lengths ranging from 7 to 193 days within the Gulf (for tag deployment details see ref. 7). Two of these tagged tuna (ID 5109026 and 5109029) were present in the GOM during April-August of 2010, overlapping temporally and spatially with the Deepwater Horizon oil spill. The tag data set contained extensive archival data enabling a thorough examination of bluefin tuna behavior in the GOM. We also use model integrated surface oil data from the Environmental Response Management Application (ERMA) as part of the National Resource Damage Assessment (NRDA) to estimate the total surface area of the GOM that was oiled, and to calculate a time series of adult Atlantic bluefin tuna habitat and spawning habitat oiled^35^,^36^. These modeled products rely on remotely sensed data for surface waters thus the effects of subsurface oil remain outside the scope of this study.

As tracking data give presence of tuna but no direct measure of absence, correlated random walks (CRWs) were used to represent a null model where tuna could move in the environment independent of oceanographic variables. For each of the 66 bluefin tuna tracks, 100 CRWs were created for use as pseudo-absences in our model framework (Supplemental Figure 1). CRWs are simulated tracks that consist of a succession of random steps, with each position generated using tag-derived turning angle and distance distributions from a specific tuna track^37^,^38^. The starting point for each CRW was the tagging location (if tagged in the GOM) or point of entry to the gulf, the initial angle of travel of the CRW matched the initial angle of the corresponding tag, each CRW position was given the same error distribution as the actual track, and each CRW has the same duration as the original tag. CRWs are not true absences because un-tagged tuna movement patterns are unknown and their distribution could potentially overlap with a null track. For each track, a random CRW was chosen from the population of CRWs to create a presence/absence dataset to fit and predict habitat likelihood, with this process repeated 60 times^38^,^39^. We also examined spatial and temporal scales of bluefin tuna movement within the GOM using residence time and first-passage time analyses in the R package adehabitat (v. 1.8.18).

Spawning dates were determined based on the diving behavior and internal temperature of archival tagged tuna (see Supplemental methods for additional information and ref. 16). Diving behavior in the GOM was characterized by deep diving as the fish entered and exited the GOM (crossing 80.5° W), and periods of shallow oscillatory diving during the night when internal temperatures increased. Telemetry-derived rapid oscillatory diving behaviors have been used to identify putative spawning, such as for skipjack tuna in the eastern Pacific^40^, bluefin tuna in the GOM^16^,^41^, and bluefin tuna in the Balearic sea^42^. While we lack direct observations of spawning from telemetry records, these oscillatory dives are uniquely found on spawning grounds and occur spatially and temporally in areas of high larval abundance^12^,^16^. Each fish with diving behavior was examined for oscillatory night diving (ref. 16) using both expert opinion of two scientists and a Bayesian model (fully described in the Supplemental methods). Putative spawning dates were combined with transiting dates to create a binary response variable with negative binomial link in the spawning likelihood generalized additive mixed model.

Modeled spawning behavior could only be calculated for the electronic tags with continuous time series records including archival tags and recovered PAT tags. Switching state-space models have been used to identify putative foraging events via distance and turn and angles^34^, and here we use both horizontal and vertical movement data with additional proxies to identify presumed spawning events. Dates and locations from state space modeled output were classified as “spawning” or “non-spawning.” We identified ten “proxy” functions to separate days hypothesized as spawning behavior from non-spawning days (see supplement). This method of combining proxies is tolerant of false detections, and allows for overlapping probability distributions. Note that this method does not require that a proxy be high or low to indicate spawning, rather it requires that the proxy values are concentrated at some point along the proxy axis during spawning. Spawning and non-spawning dates that were identified in the archival tag validation data set were also compared to visually identified spawning from a pair of human experts. These two approaches showed 87% agreement suggesting that by combining both methods we are able to robustly identify changes in behavior that typify spawning.

Remotely sensed environmental data were obtained for both actual and CRW tracks using Xtractomatic (http://coastwatch.pfel.noaa.gov/xtractomatic). The data sets included time-series of sea surface temperature (SST) and SST variability (standard deviation, SSTsd – 8 day) merged from Pathfinder and AVHRR, surface chlorophyll-a concentration (chl a – 8 day) merged from SeaWiFS and MODIS, sea surface height anomaly (SSHa – 1 day) from Aviso, SSHa variability (standard deviation, SSHsd) from Aviso, eddy kinetic energy (EKE – 1 day) derived from Aviso, vertical Ekman pumping (wekman – 8 day) derived from merged Quikscat and Ascat data, north winds (uy10 – 8 day) merged from Quikscat and Ascat data, moon phase (moon – 1 day) from NOAA, bathymetry (bathy) and rugosity (standard deviation, bathysd) both from eTOPO2. For each oceanographic variable, the geolocation error radius from each daily SSM and CRW position was used to calculate a mean value. Transformations of the environmental variables were explored to ensure data were normally distributed. A logarithmic transformation was required for Chl-a and EKE.

In order to quantify habitat use in the GOM, a binary presence/absence generalized additive mixed model (GAMM) was fit as a function of oceanographic variables incorporating a negative binomial link function. We use state space model derived geolocation error at each position to determine the radius of sampled predictor variables, which provides a probabilistic estimate of experienced environmental covariates. In addition, we assess error introduced by pseudo-track choice via AUC scores of multiple CRW combinations, and we assess model diagnostics for habitat and spawning using k-fold cross-validation with test-training datasets. Spawning likelihood models were fit using a binary spawning/non-spawning GAMM for each daily position along the track against similar oceanographic variables, and also used a negative binomial link function. GAMMs are semi-parametric models that use smoothing splines to identify the relationship between dependent variable and predictor^43^. GAMMs allow for non-linearity, non-constant variance, and non-monotonicity in contrast to more conservative linear models. The mixed model can incorporate random effects, and in our case used tag ID as a random variable to account for correlations among observations. The advantage of pairing track points with CRW points in a GAMM framework is that both absence and presence are sampled at the same spatiotemporal scales minimizing the residual autocorrelation. Various combinations of environmental data sets were included in the GAMMs based on *a priori* designation to determine the best model. The models’ explanatory and predictive power were compared using Akaike Information Criterion (AIC) and area under the curve (AUC) statistics^44^,^45^. The GAMMs were run in R (version 3.12) using the MGCV package (version 1.7–6) with cross-validation using the ROCR package (version 1.07). The GAMM model with highest AUC values and subsequently lowest AIC value was run 60 times with randomly chosen pseudo-absence tracks for each tag to quantify error derived from CRW selection. We ran model diagnostics using k-fold cross-validation in two configurations. Models were fit with randomly selected 75 training/25% test data, and subsequently each year was removed when fitting the model as test data to examine interannual predictive ability. The cross-validation process was repeated for the spawning model and a single best-fit spawning model was chosen for prediction purposes.

Environmental relationships from the best-fit GAMMs were used to predict (1) adult ABFT habitat and (2) spawning likelihood within that habitat, both as a function of ocean variables from April 1^st^ to August 26^th^, 2010 in weekly increments. Response surfaces for the time of the oil spill were aggregated by week at 0.25° x 0.25° spatial resolution. Binary cut-offs were determined using the ROCR package (version 1.0-7) in order to minimize the false positive rates and provide conservative estimates of both habitat and spawning likelihood. Modeled habitat likelihood and spawning likelihood were multiplied together to get estimates of total spawning habitat during the duration of the oil spill. Oil products were also interpolated to the nearest date to provide comparable oil extent in the Gulf of Mexico. The amount of spawning habitat in the GOM was summarized for the US EEZ for comparison with previous studies, as well as for the entire Gulf of Mexico in relation to overlap with oil layers. We calculated the percentage of habitat within the oil spill that was predicted bluefin spawning habitat in addition to the total percentage of spawning habitat that was oiled. These two metrics provide complementary statistics on the overlap between oil and tuna and secondarily quantify potential impact on spawning adult tuna in the GOM.

## Results

**Figure 1:**
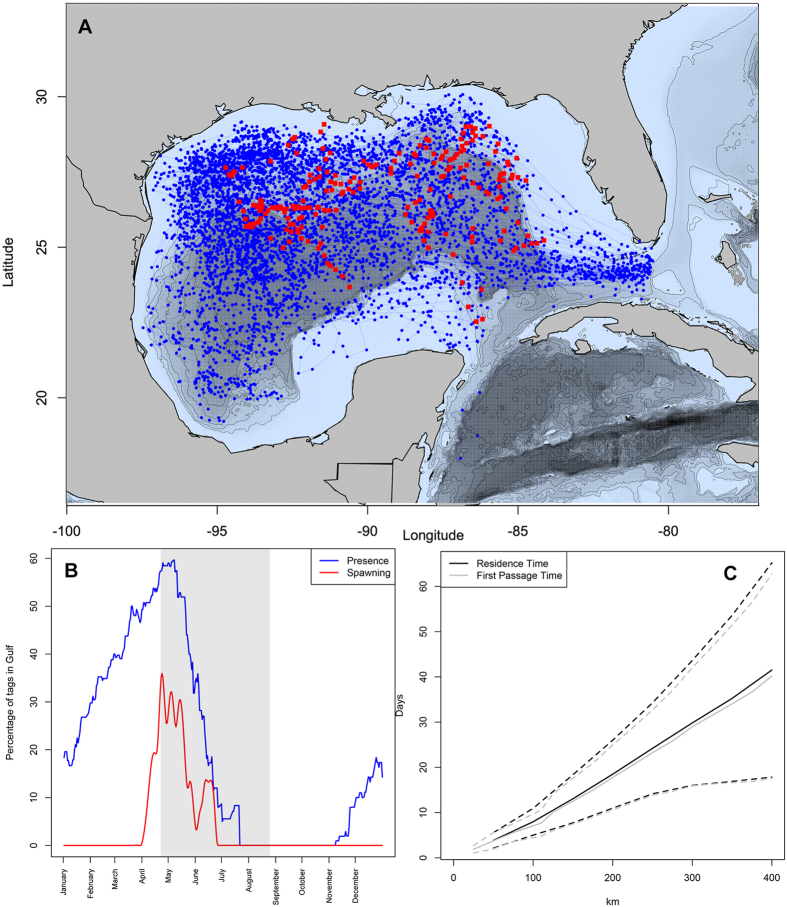
Atlantic bluefin tuna residence and spawning behavior in the Gulf of Mexico from tag data (1997–2014). (**A**) State-space modeled presence is shown in blue with tag-derived spawning events shown in red. (**B**) Temporal occupation of the gulf is shown in blue measured by percentage of tagged fish with peak percentage of fish spawning shown in red. The period of the Macondo well oil spill in 2010 is shown in grey. (**C**) Residence time and first passage time illustrate spatio-temporal scales of movement patterns of tagged bluefin tuna within the gulf. Figures were made in R v. 3.2.0: A Language and Environment for Statistical Computing, R Core Team, R Foundation for Statistical Computing, Vienna, Austria (2016) https://www.R-project.org.

Tag data from 1997–2014 indicate that the peak occupancy in the GOM for bluefin tuna (40% of tagged fish) occurs from March 15^th^ to May 31^st^ (Fig. 1A,B) which agrees with previous analyses of Atlantic bluefin tuna electronic tag data, larval data, and bycatch data^7^,^11^,^12^,^46^. Proposed spawning behavior was identified between April 1^st^ and June 28^th^ with a peak (>20% of tags with spawning) between April 15^th^ and May 20^th^ (Fig. 1B). Bluefin tuna occupied the entire GOM, while spawning was generally more restricted to the northern GOM slope waters (>23° N latitude). Residence time and first-passage time analyses for the entire GOM dataset indicated that the average scale of movement was 100 km every 4.3–10.1 days (Fig. 1C). Given that tuna could easily move out of a 100 km × 100 km grid cell within a week (Fig. 1C), each week was considered new habitat.

Models of bluefin habitat (presence vs. simulated absence) resulted in a subset of 6 environmental variables selected in the most parsimonious model (Table 1, Supplemental Table 2, Supplemental Fig. 2). The summary statistics showed that these variables remained significant across all 60 runs of the models (100%), except for SSHa and SSHsd, which were significant in 78% and 93% of the CRW iterations respectively (Table 1). The spawning model selected included 9 out of 11 terms from the full model (Supplemental Table 2). The final habitat and spawning models both had higher AUC values than the reduced model (SST, SSHa, EKE), so the best-fit models were chosen for predictive purposes to maximize our accuracy (Table 1, Supplemental Table 2, Supplemental Figures 3–5). Cross validation for 75% training/25% test with 5 folds resulted in mean AUC values of 0.789 and 0.905 for the presence/absence and spawning model respectively. Cross validation by year (13 folds) resulted in mean AUC values of 0.758 for the presence/absence, and 0.901 for the spawning model.

**Figure 2:**
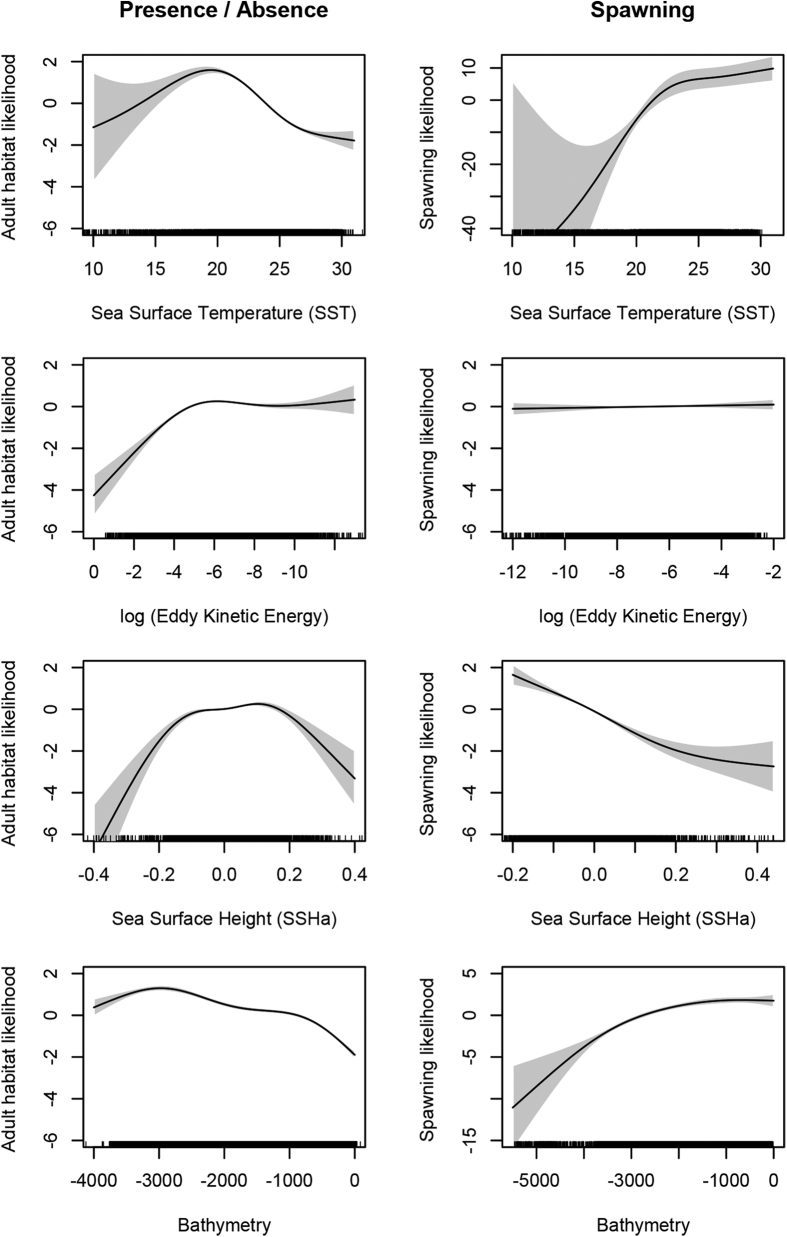
Generalized additive mixed model response curves for (**A**) presence-absence and (**B**) spawning likelihood. Positive values on the y-axis indicate increased likelihood of presence or spawning. The four most significant explanatory variables, sea surface temperature, eddy kinetic energy, sea surface height, and bathymetry are shown here, with complete GAMM splines shown in Supplemental Figs 3 and 4. Figures were made in R v. 3.2.0: A Language and Environment for Statistical Computing, R Core Team, R Foundation for Statistical Computing, Vienna, Austria (2016) https://www.R-project.org.

**Figure 3:**
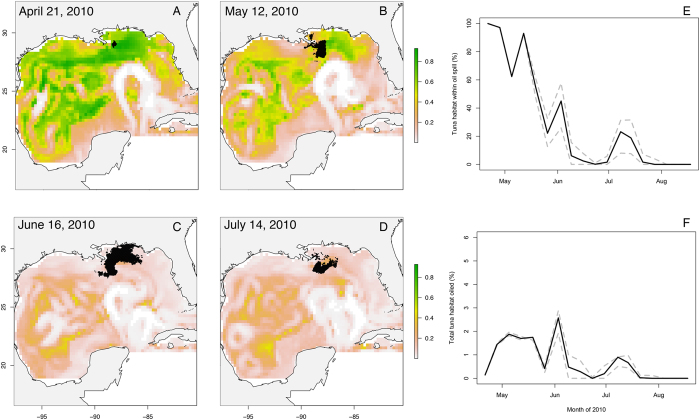
(**A–D**) Spawning habitat predictions and oil spill extent over the duration of the oil spill. Likelihood of habitat ranges from low (white and pink) to high (yellow and green). Timeseries of (**E**) the habitat contained within the oil spill (percentage of oil spill area) represents how much of the oiled extent was considered tuna habitat, while (**F**) the amount of tuna habitat oiled (percentage of total habitat) represents the how much of predicted Gulf of Mexico spawning habitat was covered in oil. Figures were made in R v. 3.2.0: A Language and Environment for Statistical Computing, R Core Team, R Foundation for Statistical Computing, Vienna, Austria (2016) https://www.R-project.org.

The models demonstrated that Atlantic bluefin tuna in the GOM had strong associations with SSTs between 15 and 25 °C, higher EKE values (>0.001 log m^2^·s^−2^), and slightly positive SSHa values (between −0.16 and 0.2 cm). Tuna also avoided shallower shelf waters with a preference for the shelf break, slope and deeper waters (Fig. 2, Supplemental Figs 2,4 and 5). Spawning habitat preferences had similar environmental drivers to total habitat with some notable exceptions. Bluefin tuna were more likely to exhibit spawning behavior at high temperatures (>22 °C), and low SSHa values (<−0.05 cm), and spawning behavior was more likely during full moons. Adult tuna avoided the loop current (higher EKE values, Fig. 2G) and warmer waters as the season progressed (higher SST values) and predicted spawning habitat also excluded the loop current (Figs 2F and 3). The combined habitat predictions showed preferred bluefin tuna habitat was most persistent in the western and northern GOM throughout the duration of the oil spill. However, the overall availability of preferred habitat decreased particularly after June 1^st^ of 2010 (Fig. 3).

Estimates of oiled spawning habitat were predicted beginning on April 21^st^, and oil was largely absent from surface waters by July 28^th^. Importantly, the timing of the oil spill directly overlapped with the maximum extent of adult bluefin tuna foraging and spawning habitat in the gulf (Fig. 3). Weekly oiled habitat ranged from 0 to 2.6% (mean 1%) of total bluefin habitat in the Gulf (Fig. 3F), and 0 to 5.5% (mean 1.8%) of habitat in the US EEZ, at the peak on June 6^th^, 2010. This corresponds to 54,000 km^2^ habitat oiled out of 941,394 km^2^ total habitat available on that date. At the same time, >70% of the extent of the oil spill was identified as preferred bluefin tuna habitat through May 12^th^ with that amount dropping to near 0% in early June (Fig. 3E). However, there was a secondary peak between July 1^st^ and July 21^st^ when tuna habitat and the oil spill overlapped again (20% of oil extent was habitat and <1% of total bluefin preferred spawning habitat was oiled). Summing the area of the predicted habitat and the oil spill overlap by week, the cumulative oiled tuna habitat was 8,086,130 km^2^ representing the potential for a significant impact on adult and potentially larval bluefin tuna in the GOM.

## Discussion

Atlantic bluefin tuna are the focus of international fisheries management and significant effort has been invested in trying to rebuild the GOM and Mediterranean populations in part due to their high economic value. Rebuilding plans have not yet resulted in recovery of the GOM population, and seasonal closures have recently been established to protect this population on the GOM spawning grounds^7^. The discrete GOM bluefin tuna population is largely fished in the Gulf of St. Lawrence, with fisheries landings over 500t per year recently^17^,^18^,^47^,^48^. Although bluefin tuna are not directly harvested by the US in the GOM, their reliance on the GOM for spawning has resulted in a significant level of bluefin tuna bycatch in yellowfin tuna longline fisheries^6^,^46^. The impacts of the DWH oil spill on this vulnerable and rebuilding population remain unknown.

The Deepwater Horizon was the largest oil spill in US history, in an area of huge economic and ecological importance^49^,^50^,^51^, yet the effects of this event on the ecology of the GOM remain unclear, especially for commercially important pelagic species such as the Atlantic bluefin tuna. Numerous pelagic species spawn in the GOM in the spring and summer months including Atlantic bluefin, yellowfin, and blackfin tunas, blue marlin, swordfish and mackerels, and consequently numerous economically important fisheries operate within the GOM^12^,^16^,^23^. We demonstrate that there was significant overlap between bluefin tuna spawning habitat (eggs, larvae and adults) and oiled surface waters in the Gulf of Mexico, up to 2.6% of their total Gulf habitat or 5.5% of their habitat within the US EEZ. Our estimates are slightly lower than those from larval surveys (5–11%), but previous calculations used preliminary oil surface layers and focused only on the northern GOM^11^. The ultimate effects of having between 2.6% of their total GOM spawning habitat to 11% of their larval habitat within the US EEZ^11^ exposed to oil are unclear, but they may have potentially important effects on the 2010 year-class of Atlantic bluefin tuna. Monitoring 2010 and future cohorts would allow assessment of whether DWH had an acute effect on bluefin tuna, although chronic impacts may take much longer to manifest^52^.

There are well documented impacts in larval tuna exposed to oil via the blocking of critical cardiac pathways^26^,^53^,^54^, yet the effects on juveniles and adults remain less clear. Experimental effects of oil on chub mackerel indicated that four days of cumulative exposure significantly impaired their metabolic rates^55^. Adult bluefin are capable of moving at a scale of ~100 km per week thus it is likely they could move to avoid direct exposure to oil for prolonged periods. Nonetheless, it is possible that even minimal exposure for fish with high gill surface areas when combined with the energetic costs of spawning and high temperatures in the GOM may have a synergistic deleterious effect on adult survival (e.g. ref. 56) or long-term reproductive output^57^. Similar effects have been observed in Prince William sound where chronic exposure inhibited reproduction and increased mortality rates for fish years after the initial spill^52^. Bluefin tuna are not obligate spawners and can skip years, although spawning can occur for multiple days and multiple bouts within a season^58^. Even though there is high variability in output and natural mortality of bluefin tuna larvae^59^, increased larval mortality due to oil exposure may further reduce the resilience of this population to continued high fishing pressure.

By examining total habitat use by Atlantic bluefin tuna and putative spawning behavior, we were able to predict spawning habitat for adults and presumably the resulting distribution of eggs and larvae^7^,^16^. Spawning events were more constrained than adult habitat, with moon phase playing an important role in these models. We found that spawning likelihood was greater in low SSHa waters, and warmer waters similar to previous findings from adult and larval data in the GOM^6^,^12^,^19^,^41^. In addition, the timing and spatial extent of spawning here matches the phenology and distribution of larval bluefin tuna in the GOM^19^, providing strong support for our use of night-time, shallow, rapid oscillatory dives as a proxy for spawning. Because tuna entered the GOM as early as November and spawning was largely in the late spring and early summer when water temperatures were warmer, the model predicts spawning at warmer temperatures compared to habitat use models (Fig. 2E,G). Tuna spawning is predicated on presence in the GOM, thus the results of the spawning model must be interpreted in concert with the results of the presence/absence model. In other words, tuna cannot spawn in a location that an adult does not occur. The preference for temperatures cooler than 25 °C in the habitat model suggests that tuna occupy cooler habitat within the GOM, but spawn preferentially later in the season and in lower SSHa waters. Similar to the findings here, bycatch data show increased catch rates at lower SSHa values and cooler temperatures, suggesting preferential use of mesoscale eddies^46^. Given tuna spawn late in the season before leaving the GOM, spawning likely occurs at an optimal temperature for the larvae, yet close to the physiological thermal limit of bluefin (Figs 1B and 2, refs 12,16 and 46) increasing their potential sensitivity to additional stressors such as oil and bycatch. Larval distribution models fit to climate projections estimated that spawning habitat in late spring would contract due to warming while early spring spawning may become more common, which substantiates these potential physiological limits of adult bluefin^20^.

The combination of increased capabilities of electronic tags^60^,^61^,^62^ with advances in statistical techniques has expanded the use of habitat models in marine conservation and management^63^,^64^. However, the dichotomy between analysis techniques for tag-based movement data and fisheries stock assessments still make combining these approaches difficult^65^,^66^. Recently, tag data have been combined with Bayesian movement models to analyze and incorporate mixing among discrete populations for use in improving international quota allocation^66^. Tag data have been integral in ecosystem based management approaches such as overlap with anthropogenic risks^31^, designation and assessment of protected areas^67^, predicted response to climate change^68^, and assessing bycatch risk in marine fisheries^69^. Moving beyond static and seasonal closures, tag data have been critical in identifying dynamic management areas such as for turtles in the central Pacific^70^,^71^ and bluefin tuna in Australia^72^. Seasonal closed areas for longlines targeting GOM yellowfin tuna have been implemented in 2014–2017 for April and May to minimize bluefin tuna bycatch^7^,^73^. Importantly, the tuna habitat model presented here could be used in either a near-real time predictive mode using satellite data or in forecast mode with forward-looking projections from ocean models to offer dynamic closures that would track key ocean features, potentially minimizing bycatch in longline fisheries^72^,^74^,^75^,^76^. Combining adult Atlantic bluefin tag data with fisheries observer bycatch data and larval sampling data would provide a holistic picture of bluefin tuna habitat use in the GOM to improve dynamic approaches to conserve this rebuilding population.

## Additional Information

**How to cite this article**: Hazen, E. L. *et al.* Quantifying overlap between the Deepwater Horizon oil spill and predicted bluefin tuna spawning habitat in the Gulf of Mexico. *Sci. Rep.* **6**, 33824; doi: 10.1038/srep33824 (2016).

## Supplementary Material

Supplementary Information

Supplementary Video 1

## Figures and Tables

**Table 1:** Generalized additive mixed model output for the selected best model (presence/absence), reduced model (presence/absence), and the selected best spawning model.

n (models)	Full model	Reduced model	Spawning model
	*60*	*60*	*1*
s(sst)	100%	100%	100%
s(log(eke))	100%	100%	—
s(ssha)	78%	49%	100%
s(sst_sd)	100%	—	—
s(ssha_sd)	93%	—	—
s(bathy)	100%	—	100%
s(log(chl))	—	—	100%
s(uy10)	—	—	100%
s(bathy)	—	—	100%
s(bathyrms)	—	—	100%
s(moonphase)	—	—	100%
*R-squared*	*0.25*	*0.13*	*0.27*
*AUC*	*0.794*	*0.703*	*0.72*
*AIC*	*54055*	*49010*	*67258*

The amount of times each term remained significant over 60 runs is represented by percentages.
