# Corrigendum: Quantifying overlap between the Deepwater Horizon oil spill and predicted bluefin tuna spawning habitat in the Gulf of Mexico

**DOI:** 10.1038/srep36689

**Published:** 2016-11-10

**Authors:** Elliott L. Hazen, Aaron B. Carlisle, Steven G. Wilson, James E. Ganong, Michael R. Castleton, Robert J. Schallert, Michael J. W. Stokesbury, Steven J. Bograd, Barbara A. Block

Scientific Reports 6: Article number: 3382410.1038/srep33824; published online: 09 22 2016; updated: 11 10 2016

The original version of this Article contained typographical errors.

In the Abstract,

“More than 54,000 km^2^ (5%) of predicted spawning habitat within the US EEZ was oiled during the week of peak oil dispersal, with potentially lethal effects on eggs and larvae”.

now reads:

“More than 13,600 km^2^ (5%) of predicted spawning habitat within the US EEZ was oiled during the week of peak oil dispersal, with potentially lethal effects on eggs and larvae”.

In the Results section,

“This corresponds to 54,000 km^2^ habitat oiled out of 941,394 km^2^ total habitat available on that date. At the same time, >70% of the extent of the oil spill was identified as preferred bluefin tuna habitat through May 12^th^ with that amount dropping to near 0% in early June (Fig. 3E). However, there was a secondary peak between July 1^st^ and July 21^st^ when tuna habitat and the oil spill overlapped again (20% of oil extent was habitat and <1% of total bluefin preferred spawning habitat was oiled). Summing the area of the predicted habitat and the oil spill overlap by week, the cumulative oiled tuna habitat was 8,086,130 km^2^ representing the potential for a significant impact on adult and potentially larval bluefin tuna in the GOM”.

now reads:

“This corresponds to 13,600 km^2^ habitat oiled out of 518,505 km^2^ total habitat available on that date. At the same time, >70% of the extent of the oil spill was identified as preferred bluefin tuna habitat through May 12^th^ with that amount dropping to near 0% in early June (Fig. 3E). However, there was a secondary peak between July 1^st^ and July 21^st^ when tuna habitat and the oil spill overlapped again (20% of oil extent was habitat and <1% of total bluefin preferred spawning habitat was oiled). Summing the area of the predicted habitat and the oil spill overlap by week, the cumulative oiled tuna habitat was 86,305 km^2^ out of 8,086,130 km^2^ total habitat representing the potential for a significant impact on adult and potentially larval bluefin tuna in the GOM”.

These errors have now been corrected in the PDF and HTML versions of the Article.

