# Supplementary material for: Quantifying overlap between the Deepwater Horizon oil spill and predicted bluefin tuna spawning habitat in the Gulf of Mexico
Source: Sci Rep. 2016 Sep 22;6:33824. doi: 10.1038/srep33824 (PMC5031980; doi:10.1038/srep33824)
Supplement: Supplementary Information [file srep33824-s1.doc]

**Quantifying overlap between the Deepwater Horizon oil spill and predicted bluefin tuna spawning habitat in the Gulf of Mexico**

Authors: Elliott L. Hazen^1,2,*^, Aaron Carlisle^1,3^, Steven G. Wilson^3^, James E. Ganong^3^, Michael R. Castleton^3^, Robert J. Schallert^3^, Michael J.W. Stokesbury^4^, Steven J. Bograd^1^, and Barbara A. Block^3^

^1^ Environmental Research Division, NOAA SWFSC, Monterey, California 93940, USA

^2^ Department of Ecology and Evolutionary Biology, University of California Santa Cruz, Santa Cruz, California 95064, USA

^3^ Hopkins Marine Station, Stanford University, Pacific Grove, California 93950, USA

^4^ Department of Biology, Acadia University, Wolfville, Nova Scotia, Canada B4P 2R6

^*^Correspondence to: Elliott.Hazen@noaa.gov

**Supplemental Methods**

The Matlab function fitcnb was used to estimate spawning days. The proxies values were used as the predictors for fitcnb and the union of two expert visual observers of spawning, Drs Wilson and Dr. Block who predicted spawning days were used as the labels for fitcnb. The kernel density method was used for each of the 10 predictors below.

1. *Water temperature at 100 meters*. The tag water temperature, from the time step with the depth closest to and not less than 100m. Examining a plot of depth versus temperature coded by the spawning “validation” training data set (fully archival tag with internal temperature, external temperature, pressure and light), we found colder water temperatures at greater depth on days human observers had visually assigned as “spawning days” in the GOM. To create the quantitative proxy function from the qualitative observations we standardized on observing temperature in the GOM at a depth of 100m. This cooling during spawning may represent a shallow thermocline from upwelling, which can occur for example in a cold core ring or when a current hits the continental slope.

2. *Light Extinction*. Normalized slope of light versus depth. The linear least squares slope was taken from the light data versus depth using only day time data during the 24h period, and only from depths 10-350m. Because the units of this light extinction vary between instruments, it was normalized by the range of all the days of the same fish in the GOM.

3. *Dawn Minimum Depth.* Minimum depth of the animal in the window starting one hour before and ending one hour after sunrise.

4. *Deepness*. 90th percentile of the 24h depth.

5. *Night Deepness*. 90th percentile of night time depth.

6. *Day Deepness*. 90th percentile of day time depth.

7. *SSM Speed*. Change in SSM location in degrees per day.

8. *Night Lingering and Zooming*. Sum of count of night time samples “lingering” or “zooming”. Lingering was defined as staying at the same depth, with an absolute change in depth less than or equal to 1 meter. Zooming was defined as diving at a high climb rate, with an absolute change in depth greater than 80 meters. Datasets were subsetted to 90 depth samples per day, and only night time samples were counted.

9. *Stable Time at Depth* *Histograms.* Normalized sum of RMS of each day's depth histogram versus the previous and next day's. We made histograms of depth, with bins ranging from -10m to 388m with an interval of 2 meters. -10m was chosen because the data for this analysis had not been zero offset corrected, so the surface depth is less than 0 on some days. The root mean square of the difference of each day's histogram compared to the previous day was added to the RMS of the each day compared to the next. Finally, this sum was normalized by the range of this sum for all days of the same fish in the Gulf.

10. *Stable time at Temperature Histograms*. Same as discussed above n Stable Time at Depth histograms, but based on Time at Temperature. Binning variable was temperature from 1 to 30 degrees with an interval of 1 degree. The in-gulf portion was selected from each track, and these selected portions were combined into one dataset.

**Supplemental Figures and Tables**


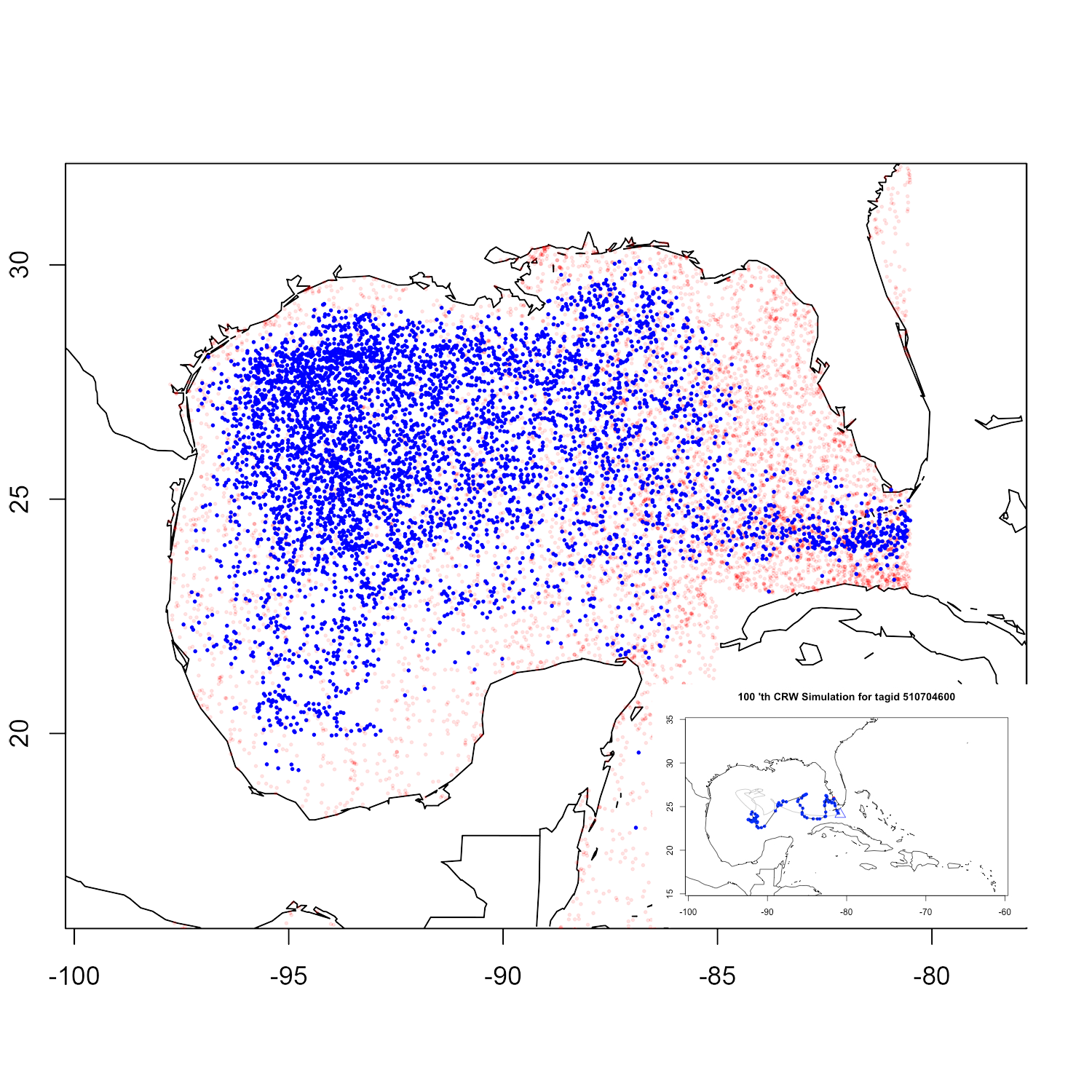


Supplemental Figure 1. Correlated random walk simulation for Tag ID 510704600 shown in blue with the state-space modeled actual track shown in grey. Figures were made in R v. 3.2.0: A Language and Environment for Statistical Computing, R Core Team, R Foundation for Statistical Computing, Vienna, Austria (2016) [https://www.R-project.org](https://www.r-project.org/).

Supplemental Figure 2. Histograms of environmental variables sampled along tracks and pseudotracks, the correlated random walk simulated absences. Figures were made in R v. 3.2.0: A Language and Environment for Statistical Computing, R Core Team, R Foundation for Statistical Computing, Vienna, Austria (2016) [https://www.R-project.org](https://www.r-project.org/).

Supplemental Figure 3. GAMM response curves for chosen presence / absence model. Response is on the y-axis with the predictor variable range on the x-axis (actual data shown as rug plots). Figures were made in R v. 3.2.0: A Language and Environment for Statistical Computing, R Core Team, R Foundation for Statistical Computing, Vienna, Austria (2016) [https://www.R-project.org](https://www.r-project.org/).

Supplemental Figure 4. GAMM response curves for chosen spawning likelihood model. Response is on the y-axis with the predictor variable range on the x-axis (actual data shown as rug plots). Figures were made in R v. 3.2.0: A Language and Environment for Statistical Computing, R Core Team, R Foundation for Statistical Computing, Vienna, Austria (2016) [https://www.R-project.org](https://www.r-project.org/).


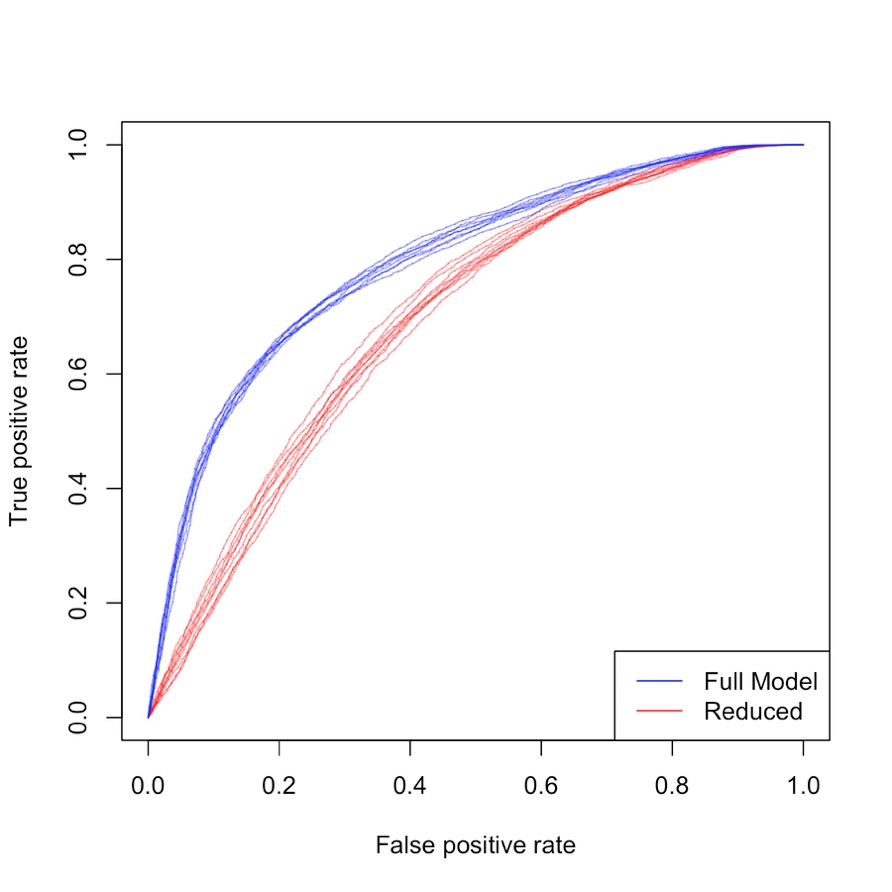


Supplemental Figure 5. A) Receiver operating characteristic (ROC) curves for 60 full and reduced models, and B) ROC curves for the best spawning model. ROC curves indicate the true positive rate (y-axis) relative to the false positive rate (x-axis) for use in determining model predictive capacity (area under the curve statistic) and binary habitat cut-offs. Figures were made in R v. 3.2.0: A Language and Environment for Statistical Computing, R Core Team, R Foundation for Statistical Computing, Vienna, Austria (2016) [https://www.R-project.org](https://www.r-project.org/) using the ROCR package v1.0-5 (https://rocr.bioinf.mpi-sb.mpg.de/).

Supplemental Video. Weekly spawning habitat predictions and oil spill extent from April 20^th^- August 26^th^. Video frames were made in R v. 3.2.0: A Language and Environment for Statistical Computing, R Core Team, R Foundation for Statistical Computing, Vienna, Austria (2016) [https://www.R-project.org](https://www.r-project.org/) and animated in Adobe Photoshop CS6 (http://www.adobe.com/).

Supplemental Table 1. Summary of archival and pop-up satellite archival tags that entered the Gulf of Mexico for more than 7 days. Spawning dates that were detected via SSSM and expert identification are shown in the last column.

| **Supplemental Table 1.** Summary of archival and pop-up satellite archival tags that entered the Gulf of Mexico for more than 7 days. Spawning dates that were detected via SSSM and expert identification are shown in the last column. | | | | | |
| --- | --- | --- | --- | --- | --- |
| PTT | Year | Date of Entry | Date of Exit | Days in GOM | Spawning detected? |
| 5100133 | 1999 | 6/1/99 | 6/25/99 | 25 | TRUE |
| 5101333 | 2001 | 4/16/01 | 5/31/01 | 46 | FALSE |
| 5101336 | 2001 | 4/24/01 | 5/28/01 | 35 | TRUE |
| 5102412 | 2002 | 4/10/02 | 4/22/02 | 13 | TRUE |
| 5102413 | 2002 | 4/13/02 | 5/20/02 | 38 | FALSE |
| 5102416 | 2002 | 4/19/02 | 5/23/02 | 35 | TRUE |
| 5102418 | 2002 | 4/20/02 | 5/26/02 | 37 | FALSE |
| 5102419 | 2002 | 4/21/02 | 4/29/02 | 9 | TRUE |
| 5103535 | 2003 | 5/16/03 | 6/18/04 | 69 | TRUE |
| 5104457 | 2004 | 4/19/04 | 6/13/04 | 56 | FALSE |
| 5104456 | 2004 | 5/24/04 | 6/19/04 | 27 | TRUE |
| 5104527 | 2004 | 11/22/04 | 2/12/05 | 41 | TRUE |
| 5105027 | 2005 | 2/18/05 | 2/18/05 | 1 | FALSE |
| 5107043 | 2008 | 1/16/08 | 3/31/08 | 76 | TRUE |
| 5107037 | 2008 | 2/7/08 | 3/20/08 | 43 | TRUE |
| 5107046 | 2008 | 2/7/08 | 4/23/08 | 77 | TRUE |
| 5108017 | 2009 | 1/14/09 | 6/4/09 | 141 | TRUE |
| 5108023 | 2009 | 1/21/09 | 1/26/09 | 6 | TRUE |
| 5108020 | 2009 | 3/9/09 | 5/24/09 | 77 | TRUE |
| 5108021 | 2009 | 3/17/09 | 6/12/09 | 84 | TRUE |
| 5108024 | 2009 | 3/19/09 | 5/22/09 | 65 | TRUE |
| 5109027 | 2009 | 12/23/09 | 3/27/10 | 95 | TRUE |
| 5109026 | 2010 | 2/21/10 | 5/10/10 | 79 | TRUE |
| 5109029 | 2010 | 4/5/10 | 5/19/10 | 45 | TRUE |
| 5110070 | 2010 | 11/8/10 | 2/2/11 | 87 | TRUE |
| 5110076 | 2010 | 11/12/10 | 1/4/11 | 54 | TRUE |
| 5110062 | 2010 | 11/24/10 | 3/5/11 | 102 | TRUE |
| 5110078 | 2010 | 12/16/10 | 2/28/11 | 74 | TRUE |
| 5110075 | 2010 | 12/28/10 | 6/30/11 | 185 | TRUE |
| 5110063 | 2011 | 1/2/11 | 5/5/11 | 124 | TRUE |
| 5110065 | 2011 | 1/11/11 | 4/14/11 | 94 | FALSE |
| 5110067 | 2011 | 1/13/11 | 3/25/11 | 72 | TRUE |
| 5110073 | 2011 | 1/27/11 | 2/3/11 | 8 | TRUE |
| 5110079 | 2011 | 2/20/11 | 3/28/11 | 37 | TRUE |
| 5110074 | 2011 | 2/27/11 | 5/25/11 | 88 | TRUE |
| 5111023 | 2011 | 11/19/11 | 12/6/11 | 14 | TRUE |
| 5111016 | 2011 | 11/23/11 | 5/27/12 | 187 | FALSE |
| 5111033 | 2011 | 11/24/11 | 5/19/12 | 178 | TRUE |
| 5111022 | 2011 | 12/7/11 | 4/3/12 | 119 | TRUE |
| 5111025 | 2011 | 12/15/11 | 5/9/12 | 147 | TRUE |
| 5111024 | 2011 | 12/22/11 | 4/2/12 | 59 | TRUE |
| 5111050 | 2012 | 1/1/12 | 2/6/12 | 11 | TRUE |
| 5111027 | 2012 | 1/13/12 | 5/6/12 | 115 | TRUE |
| 5111046 | 2012 | 1/15/12 | 3/25/12 | 71 | TRUE |
| 5111045 | 2012 | 1/19/12 | 6/1/12 | 135 | TRUE |
| 5111046 | 2012 | 1/20/12 | 5/11/12 | 113 | FALSE |
| 5111034 | 2012 | 3/11/12 | 5/19/12 | 70 | TRUE |
| 5111026 | 2012 | 3/18/12 | 5/27/12 | 71 | TRUE |
| 5111052 | 2012 | 3/30/12 | 5/7/12 | 39 | FALSE |
| 5112037 | 2012 | 11/29/12 | 6/10/13 | 194 | FALSE |
| 5112041 | 2012 | 12/4/12 | 6/7/13 | 186 | FALSE |
| 5112044 | 2012 | 12/10/12 | 12/23/12 | 14 | FALSE |
| 5112033 | 2012 | 12/20/12 | 5/7/13 | 93 | FALSE |
| 5112039 | 2012 | 12/23/12 | 5/14/13 | 143 | FALSE |
| 5112035 | 2013 | 2/24/13 | 7/20/13 | 147 | TRUE |
| 5112038 | 2013 | 3/12/13 | 5/1/13 | 51 | FALSE |
| 5112030 | 2013 | 4/2/13 | 6/10/13 | 70 | FALSE |
| 5112028 | 2013 | 5/25/13 | 5/25/13 | 1 | FALSE |
| 5113015 | 2013 | 11/24/13 | 5/14/14 | 172 | TRUE |
| 5113033 | 2013 | 12/1/13 | 6/11/14 | 193 | FALSE |
| 5113016 | 2013 | 12/13/13 | 6/4/14 | 84 | TRUE |
| 5113022 | 2013 | 12/14/13 | 4/29/14 | 121 | FALSE |
| 5113024 | 2013 | 12/24/13 | 4/16/14 | 89 | FALSE |
| 5113017 | 2014 | 1/20/14 | 2/13/14 | 25 | FALSE |
| 5113031 | 2014 | 2/3/14 | 6/3/14 | 121 | TRUE |
| 5113029 | 2014 | 2/13/14 | 5/19/14 | 96 | TRUE |

| Supplemental Table 2. Generalized additive mixed model parameters for A) reduced model, B) full model, and C) spawning model. | | | | | |
| --- | --- | --- | --- | --- | --- |
| **Presence / Absence Reduced** | | |  |  |  |
|  | **edf** | **Ref.df** | **F** | **p-value** | **percentage significant** |
| s(sst) | 3.896 | 4.000 | 188.444 | 0.000 | 100% |
| s(log(eke)) | 3.792 | 4.000 | 26.374 | 0.000 | 100% |
| s(ssha) | 1.710 | 4.000 | 6.279 | 0.236 | 49% |
|  |  |  |  |  |  |
| rsq = 0.13 | AIC = 49010 | AUC = 0.703 |  |  |  |
|  |  |  |  |  |  |
| **Presence / Absence Full** | |  |  |  |  |
| s(sst) | 3.744 | 4.000 | 187.175 | 0.000 | 100% |
| s(log(eke)) | 3.796 | 4.000 | 43.106 | 0.000 | 100% |
| s(ssha) | 2.604 | 4.000 | 11.537 | 0.100 | 78% |
| s(sst_sd) | 3.727 | 4.000 | 129.054 | 0.000 | 100% |
| s(ssha_sd) | 2.650 | 4.000 | 11.693 | 0.030 | 93% |
| s(bathy) | 3.935 | 4.000 | 83.098 | 0.000 | 100% |
|  |  |  |  |  |  |
| rsq = 0.25 | AIC = 54055 | AUC = 0.794 |  |  |  |
|  |  |  |  |  |  |
| **Spawning model** | |  |  |  |  |
| s(sst) | 2.859 | 4.000 | 34.940 | 0.000 |  |
| s(log(chl)) | 3.597 | 4.000 | 52.452 | 0.000 |  |
| s(log(eke)) | 0.326 | 4.000 | 0.120 | 0.219 |  |
| s(ssh) | 2.639 | 4.000 | 31.883 | 0.000 |  |
| s(uy10) | 3.713 | 4.000 | 17.335 | 0.000 |  |
| s(bathy) | 3.128 | 4.000 | 35.265 | 0.000 |  |
| s(bathyrms) | 3.488 | 4.000 | 8.372 | 0.000 |  |
| s(moonphase) | 2.289 | 4.000 | 53.286 | 0.000 |  |
| s(lon) | 2.584 | 4.000 | 30.264 | 0.000 |  |
|  |  |  |  |  |  |
| rsq = 0.27 |  | AUC = 0.72 | AIC = 67258 |  |  |
