# Supplementary figures and images for: Quantifying overlap between the Deepwater Horizon oil spill and predicted bluefin tuna spawning habitat in the Gulf of Mexico
Source: Sci Rep. 2016 Sep 22;6:33824. doi: 10.1038/srep33824 (PMC5031980; doi:10.1038/srep33824)

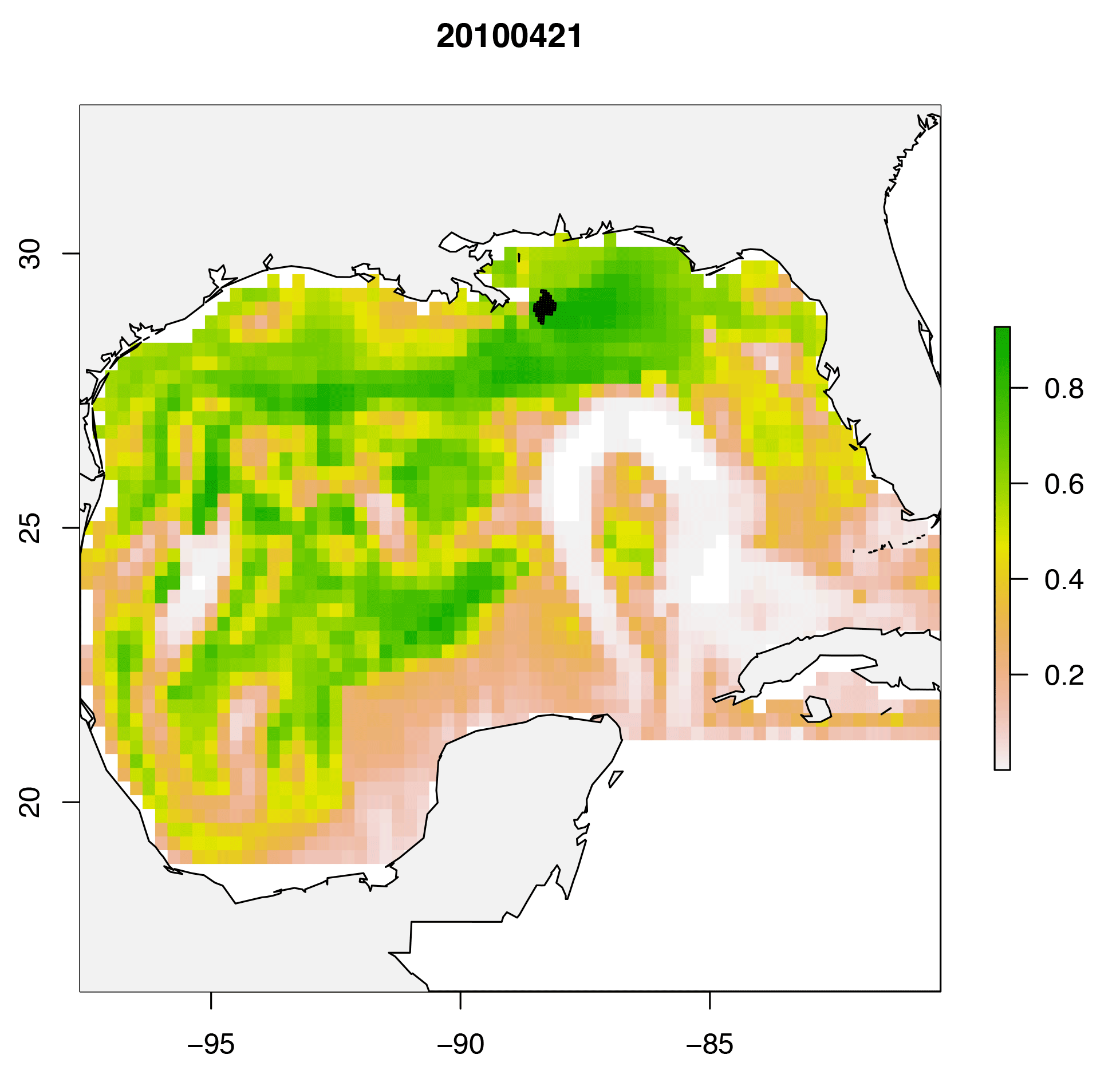

Supplement: Supplementary Video 1 [file srep33824-s2.gif]
